# A multispecies competitive nanobody-based ELISA for the detection of antibodies against hepatitis E virus

**DOI:** 10.1038/s41598-023-41955-z

**Published:** 2023-09-18

**Authors:** Lorena Paola Arce, María Florencia Pavan, Marina Bok, Silvina Elena Gutiérrez, Silvia Marcela Estein, Agostina Tammone Santos, Walter Ezequiel Condorí, Marcela María Uhart, Viviana Parreño, María Guadalupe Vizoso-Pinto, Lorena Itatí Ibañez

**Affiliations:** 1 Infection Biology Laboratory, Faculty of Medicine and Instituto Superior de Investigaciones Biológicas (INSIBIO), CONICET-UNT, T4000ILI Tucumán, Argentina; 2 Laboratorio de Ingeniería de Anticuerpos, Instituto de Química, Física de los Materiales, Medio ambiente y Energía (INQUIMAE-CONICET), C1428EGA Ciudad Autónoma de Buenos Aires, Argentina; 3 IncuINTA, Instituto de Virología, Instituto Nacional de Tecnología Agropecuaria (INTA), 1686 Husrlingham, Argentina; 4 Universidad Nacional del Centro de la Provincia de Buenos Aires, Facultad de Ciencias Veterinarias, Núcleo SAMP, Centro de Investigación Veterinaria de Tandil (UNCPBA-CICPBA-CONICET), B7000GHG Tandil, Buenos Aires Argentina; 5 One Health Institute, School of Veterinary Medicine, University of California Davis, Davis, CA 95616 USA

**Keywords:** Viral infection, Assay systems, Viral hepatitis

## Abstract

The hepatitis E virus (HEV) is an emergent zoonotic virus causing viral hepatitis worldwide. Clinically, hepatitis E is not easily distinguished from other types of acute viral hepatitis. There is a need for HEV diagnostic assays to detect and prevent interspecies transmission among susceptible populations. Nanobodies (Nbs) are expressed recombinantly in different systems, produced with high yields, and have superior physicochemical properties compared with conventional antibodies (Ab). Several Nbs against ORF2, the capsid protein and main antigen, were selected and produced in *E. coli.* Nb39 and Nb74 specifically recognized HEV ORF2 (genotypes 3 and 4). A competitive ELISA (cELISA) was developed and validated using a reference panel of human (n = 86) and swine sera (n = 116) tested in comparison with a commercial kit. The optimal cutoff values determined by ROC analysis were 69.16% (human) and 58.76% (swine); the sensitivity and specificity were high: 97.4% (95% CI 86.5–99.5%) and 95.8% (95% CI 86.0–98.8%) for human vs. 100% (95% CI 93.5–100%) and 98.3% (95% CI 91.0–99.7%) for swine. Further, the cELISA detected total anti-HEV antibodies in wild boar, deer, and mice. To our knowledge, this is the first report of production of Nbs against HEV-3 ORF2 for diagnostic purposes.

## Introduction

The Hepatitis E virus (HEV) is an emerging pathogen, which is the leading cause of acute icteric hepatitis in developing countries. According to the World Health Organization (WHO), 20 million HEV infections and 3.3 million symptomatic cases of hepatitis E are estimated each year worldwide^1^. The high seroprevalence found in industrialized countries and in immunocompromised patients who develop chronic disease, support the idea that hepatitis E has evolved from an enteric self-limiting illness to a multifactorial and chronic disorder^2^.

Usually, the infection resolves within 2–6 weeks but occasionally it develops into a serious disease known as fulminant hepatitis, which can be fatal in 0.5–4% of the patients^3^. The acute form of the infection is more severe in pregnant women, organ transplant recipients, oncologic patients, HIV-positive patients or those with preexistent liver diseases^4–7^. Chronic hepatitis may develop in certain risk groups such as patients undergoing a solid organ transplantation, with immune deficiencies or receiving immunosuppressive therapies^8^. The HEV ability to replicate in tissues other than the liver, such as placenta, kidney, brain, small intestine and spleen, has been related to its extra-hepatic manifestations^9^, although it is still a question of debate if they are a consequence of the replication itself or of the immune response. The most common manifestations are neuralgic amyotrophy (NA), Guillain–Barré syndrome, encephalitis, renal insufficiency, and blood alterations^9^.

HEV belongs to the family *Hepeviridae*, genera *Orthohepevirus,* genus: *Paslahepevirus*^10^. HEV is a single-stranded positive-sense RNA virus that encodes for three partially-overlapping open reading frames (ORF), ORF1 to ORF3, and three untranslated regions (UTR). ORF2 encodes the virus structural capsid protein whose main function is to protect the integrity of the viral genome and is the main antigen for the host humoral immune response^11^.

In the past, HEV was limited to endemic areas and was mainly transmitted by the oral-fecal route, but currently it has also been recognized as a zoonotic viral hepatitis^12^. Further, the virus can also be transmitted by blood transfusion and organ transplantation. Four genotypes can infect humans: HEV-1 and HEV-2 are waterborne, whereas HEV-3 and HEV-4 are zoonotic^13,14^. However, the last two have also been detected in river, and waste and sewage water in Europe and South America^15^. The main animal reservoirs of HEV are domestic pigs and wild boars, but several other species including domestic and wild animals such as cats, dogs, deer, ferrets, and rodents can also be infected by HEV^16,17^. The most common transmission routes of the zoonotic genotypes to humans are occupational exposure, consumption of contaminated undercooked meat and meat-derived products from infected pigs, or the consumption of fecal contaminated water, fruits, and vegetables grown on fields fertilized with manure of infected pigs^18–20^.

Data about prevalence of HEV in the Americas suggest that there is an increasing incidence of hepatitis E in the continent and that HEV-3 is the main circulating genotype^21,22^. Something similar happened before in Europe: the awareness of the disease and increment in testing revealed that the HEV infection was more common than expected^23^. Approximately 40% of the pigs from slaughterhouses in the U.S. tested positive for HEV antibodies^24^. In Brazil, serological surveys in swine showed high prevalence rates ranging from 88.4 to 97.3%^25,26^. In contrast, the seroprevalence observed in pigs from Argentina, Chile, and Uruguay vary broadly from 0.6 to 58%^27,28^.

Some studies have shown that the HEV viremic rate at the time of slaughter is relatively high and puts the food chain at risk: the blood containing HEV virions may contaminate pork meat and lead to possible foodborne infections^29^. The strong interdependence between the environment, humans, and animal health has led to the concept of One Health. Considering this notion, together with the evidence of the exposure and susceptibility of different animals to HEV, it becomes clear that more knowledge is needed about the distribution, the reservoirs, and the transmission routes of HEV. In this sense, there is still a need for HEV diagnostic assays, not only for swine and human samples, but also for other species, to predict and take measures to prevent interspecies transmission among susceptible populations.

Clinically, hepatitis E is not easily distinguished from other types of acute viral hepatitis. Its diagnosis is recommended mainly considering the epidemiology in endemic areas, under suspicion of contaminated water or when other hepatitis viruses have been excluded. Hepatitis E can be diagnosed by detecting antibodies, such as specific anti-HEV immunoglobulin M (IgM) by ELISA or rapid tests, or by reverse transcriptase polymerase chain reaction (RT-PCR) to detect the RNA of the HEV^6,30,31^. The ELISA is a versatile technique commonly used in diagnostic laboratories because it does not need special equipment except for an ELISA reader. However, in several Latin American countries kits are imported from abroad and therefore they are very expensive for the public health system. On the other hand, RT-PCR diagnostic requires specialized laboratories and trained personnel in molecular biology.

HEV testing in animals has not yet been regulated in most countries. In the U.S. there is a commercial kit available for swine based on the ORF2 and ORF3 proteins (PrioCHECK^®^ HEV Antibody ELISA)^32^. Researchers have used kits intended for humans and changed the specific conjugated antibody for swine^33^. Other detection kits are based on a sandwich ELISA and detect anti-HEV antibodies in different species using ORF2 or a synthetic peptide as both capture and detection reagent, the latter conjugated with Horse Radish Peroxidase (HRP)^34^.

In recent years, Nbs have emerged as an alternative to monoclonal antibodies or their fragments to generate reagents for the diagnosis of various pathogens^35,36^. These proteins, obtained mostly from camelids, have unique properties such as small size, thermal and chemical stability, good affinity and selectivity, and can be easily modified, and produced at low cost^35^. Nbs have been used to develop different types of diagnostic tests, including virus detection based on molecular imaging, ELISA, lateral flow immunochromatography, and immunosensors, among others^37^. Nbs against the HEV-4 ORF2 protein protected rabbits from HEV infection^38^, but they have not been tested for diagnostic purposes. Finally, a competitive ELISA (cELISA) based on Nbs coupled to HRP was recently developed to detect antibodies against avian hepatitis E virus^39^.

In order to contribute to the One Health approach and taking advantage of the properties of Nbs, we developed a low-cost competitive ELISA based on Nbs that recognize HEV-3 ORF2 protein for diagnosis of HEV infection in multiple animal species and humans.

## Results

### Construction of an immune Nbs-library

**Figure 1:**
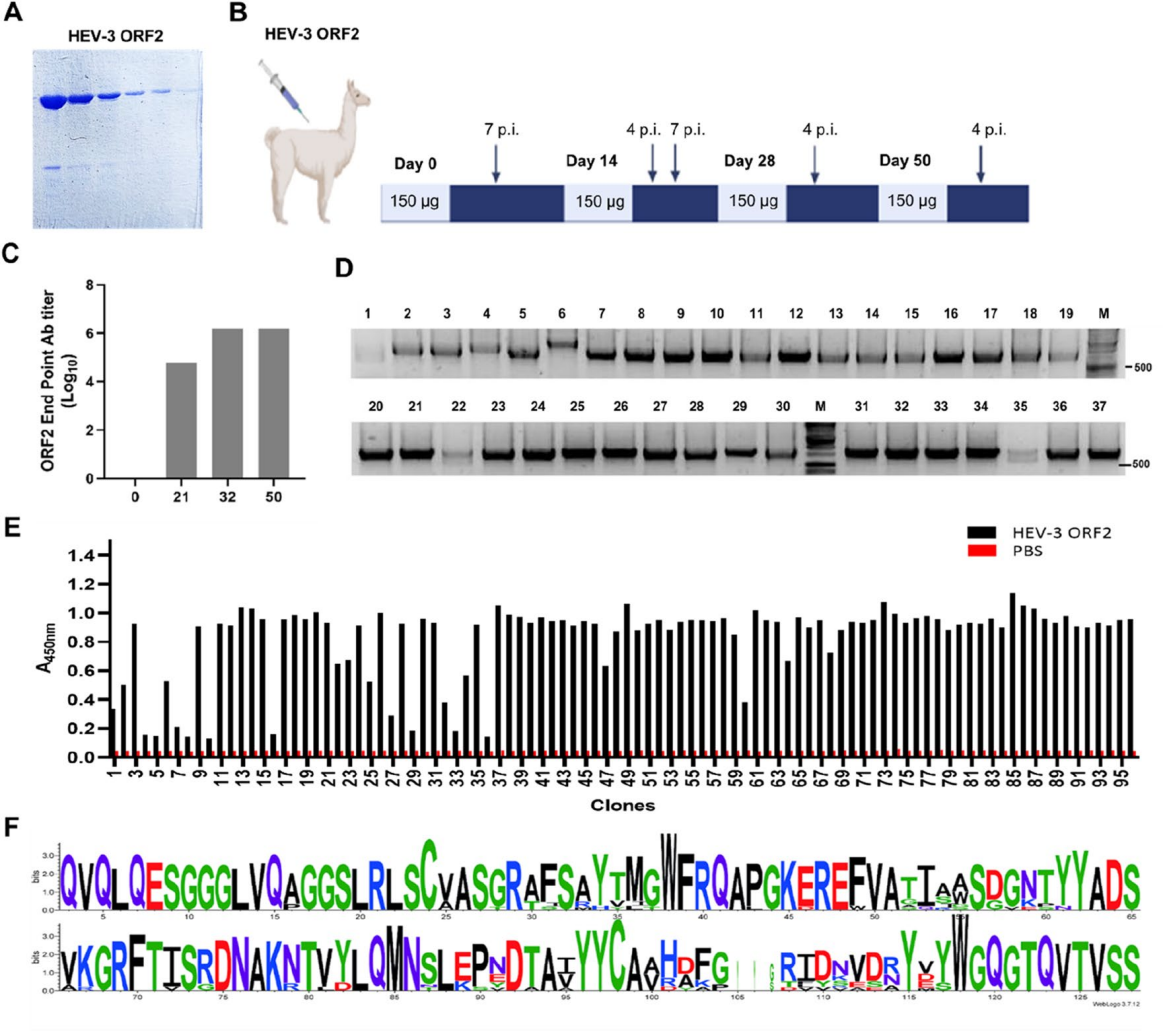
Llama immunization and screening of Nbs against the recombinant HEV-3 ORF2 protein. (**A**) SDS-PAGE and Coomassie blue staining showing the expression and purification of the recombinant HEV-3 ORF2 protein. (**B**) Schematic representation of the llama immunization protocol depicting boosting, antigen dose and blood extraction time (*p.i*. post immunization). (**C**) Antibody titer against HEV-3 ORF2 in llama sera after immunization, a maximum response is reached at PID 32. (**D**) Agarose gel showing the ~ 700 bp PCR fragments corresponding to VHHs of variable size amplified from randomly picked individual colonies. (**E**) Identification of positive clones that bind specifically to the HEV-3 ORF2 protein by ELISA, 95 periplasmic extracts from positive colonies and from a control colony were tested. (**F**) Sequence logo plot using 12 Nbs sequences (WebLog3). High variability can be observed on the AA composition of the CDR3 domains.

The recombinant HEV-3 ORF2 protein was expressed in a soluble form (66 kDa) and purified using a Ni–NTA resin^21^. According to the SDS-PAGE, the HEV-3 ORF2 protein was obtained with high purity (Fig. 1A). A llama was immunized with this protein following an immunization protocol described in Fig. 1B. Total IgG specific antibodies for HEV-3 ORF2 were evaluated by ELISA on pre-immune and post-immune sera obtained after each immunization. A specific humoral immune response was observed at post immunization day 21 (PID 21) and the anti-HEV-3 ORF2 antibody titer reached a maximum of 1,562,500 at PID 32 (Fig. 1C, end point titer), indicating that the antigen induced a good immune response in the llama. Briefly, as previously explained in the material and methods section, the total RNA extracted from lymphocytes was reversed transcribed into cDNA, then, the VHH genes (~ 400 bp) were amplified by nested-PCR (Supplementary Fig. S1A–C). Cloned VHH gene fragments were inserted into a pMECS-GG phagemid vector and a Nb-library of 1.8 × 10^9^ individual colonies was constructed. The analysis of 37 randomly picked colonies by colony PCR revealed that 100% of the phagemid plasmids contained an insert of approximately 700 bp encoding the VHH genes (Fig. 1D).

### Screening and identification of Nbs against the HEV-3 ORF2 protein

Specific Nbs against the HEV-3 ORF2 protein were selected after infecting bacteria of the Nb-library with a helper phage according to the standard rescue protocol^40^. A phage library of 3 × 10^12^ CFU/ml was obtained and used for panning. The selection of specific Nb-phages was performed after the passive coating of HEV-3 ORF2 recombinant protein in the wells of a 96-well microtiter plate. After addition and incubation of the phage library, increased number of wash steps were done after each round of panning to remove unbound phage particles. To select specific HEV-3 ORF2 Nb-phages, two successive elution methods, trypsin treatment and addition of exponentially growing bacteria, were applied as described in “Methods” section. As the selection rounds progressed, it was possible to detect a greater number of anti-HEV-3 ORF2 positive clones (Fig. 1E; Supplementary Fig. S2A). Ninety-five colonies, plus 1 colony of non-infected TG1 cells as a negative control, were picked after 3 rounds of panning and 2 elution strategies. Nanobody expression was induced with IPTG and periplasmic extracts (PE) were obtained to perform a PE-ELISA. Eighty-six clones that specifically bind to the HEV-3 ORF2 protein were identified (Fig. 1E). All periplasms were also tested on ELISA plates coated only with PBS; no positive clones were detected confirming that positive clones were specific for the protein of interest (Fig. 1E; Supplementary Fig. S2A). After analysis of the plasmids from positive clones with the frequently cutting restriction enzymes *Hinf*I (data not shown), 15 clones were selected and sequenced. A sequence logo plot was constructed considering only unique VHH sequences (12 clones). As expected, the residue conservation was high in the framework regions (FRs) and more variable on the complementarity-determining regions (CDRs) (Fig. 1F). High variability in length and amino acid (AA) composition can be observed especially in the CDR3 (Table 1, column 4 and 5).

**Table 1:**
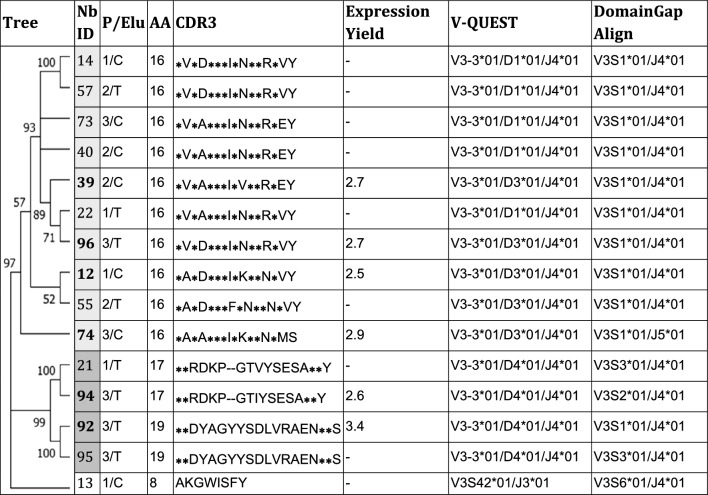
Phylogenetic analysis, selection strategy, CDR3 characteristic, yield, repertoire diversity, and germline origin of HEV-3 ORF2 nanobodies.

*Nb* nanobody, *ID* identification, *Elu* elution, *1* first round of panning, *2* second round of panning, *3* third round of panning, *C* cellular elution, *T* trypsin elution, *AA* amino acids in the CDR3.

### Genetic characterization and comparison of Nbs sequences

A phylogenetic tree was constructed with the Nbs sequence information using MEGA version 11. As Table 1 shows, Nbs can be divided into two main groups, most Nbs from the upper cluster (highlighted in light grey, supported with a 97% bootstrap value) share similar CDR3 sequences (identical residues between clones are marked by (*) suggesting the same B-cell origin. In contrast, a higher variability was seen in the lower cluster (highlighted in dark grey, supported with a 99% bootstrap). Two Nbs in this cluster contained 2 extra AA on the CDR3. Nanobody 13 possessing the shorter CDR3, was able to bind ORF2 (as shown by the ELISA results), however it contains features of a VH (variable domain of conventional antibodies), not a VHH, so it was excluded from further studies. ORF2-specific Nbs were selected from all rounds of panning (P) and with both elution (Elu) strategies, trypsin (T) treatment and cellular (C) elution (Table 1, column 3).

The germline origin was analyzed by IMGT/V-QUEST and IMGT/DomainGapAlign. The former provides information of the variable (V), joining (J), and diversity (D) gene segments, and data of *vicugna pacos* (alpaca) nucleotide sequences. The latter program allows comparison with *lama glama *(*llama*) protein sequences, but only provides information on V and J segments. We found, as expected, that Nb13 comes from a completely different set of VDJ genes, V3S42*01/J3*01 (Table 1, column 7) and V3S6*01/J4*01 (Table 1, column 8), according to V-QUEST and to DomainGapAlign, respectively. Variable segment V3-3 and allele *01 were identified for every Nb in the light grey cluster, while J segment was J4 for most Nbs except for Nb74, and D gene segments were classified as D1 or D3 according to the V-Quest program. When analyzing data on the DomainGapAlign program, all Nbs in this cluster shared the V3S1*01/J4*01V and J segments. For the lower cluster, V-Quest retrieved the V3-3*01/D4*01/J4*01 information for all clones. The DomainGapAlign found more variability in the V segments, V3S1, V3S2 or V3S3, whereas a similar J segment was shared. Considering this analysis and the variability of the CDRs sequences, we continued working with 10 Nbs, namely Nb12, Nb21, Nb22, Nb39, Nb40, Nb55, Nb74, Nb92, Nb94, and Nb96.

### Expression and purification of recombinant anti-HEV-3 ORF2 Nanobodies

The *E. coli* WK6 strain was transformed with pMECS-Nb vectors to express the recombinant Nbs. Ten selected Nbs were expressed and proteins of the expected size, approximately 15 kDa, were observed (Supplementary Fig. S3A). Six Nbs (highlighted in bold in Table 1, column 2), selected based on their yield and CDR3 AA composition (Table 1, column 5), were scaled up, purified, and further characterized (Supplementary Fig. S3B). Protein expression yields were in the 2.5–3.4 mg/L range (Table 1, column 6).

### Selection and characterization of nanobodies against HEV-3 ORF2 for diagnostic purposes

**Figure 2:**
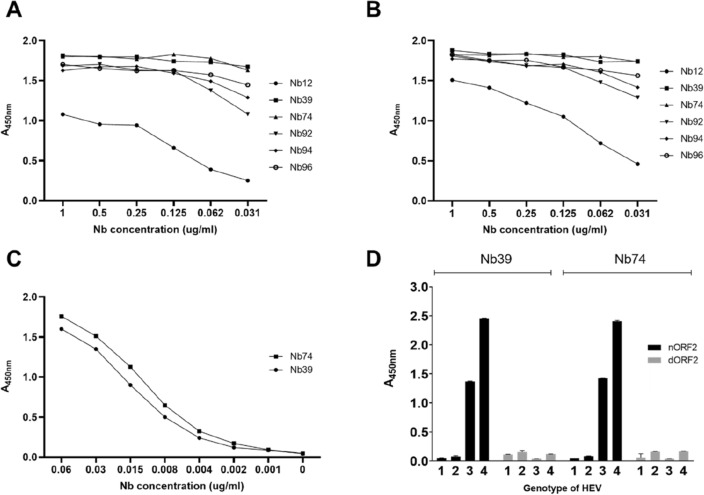
Characterization of Nbs against HEV-3 ORF2 protein. (**A**,**B**) An ELISA was performed to select the most suitable Nbs able to bind to the ORF2 protein at 2 different concentrations. A 96-well microtiter plate was coated with 0.62 (**A**) and 1.25 μg/ml (**B**) of ORF2 protein, respectively. (**C**) Further characterization of the 2 best Nbs to develop the immunoassay. A 96-well microtiter plate was coated with 0.62 μg/ml and incubated with different concentrations of Nb39 and Nb74. (**D**) Binding capacity of Nb39 and Nb74 to different form, native (nORF2) vs. denaturized (dORF2), of ORF2 proteins from 4 HEV genotypes.

The selected Nbs were analyzed by ELISA to choose the optimal Nb to develop a competitive ELISA (cELISA). A 96-well microtiter plate was coated with 2 concentrations of the antigen corresponding to the HEV-3 ORF2 protein and then incubated with different dilutions of the different Nbs. At a concentration of 0.031 μg/ml, the Nb39 and Nb74 showed the highest absorbance values at comparable dilutions, independently of the antigen concentration used (Fig. 2A,B). Then, Nb39 and Nb74 were evaluated at lower concentrations using 0.62 μg/ml ORF2. Similar absorbance was obtained for both Nbs (Fig. 2C).

We also wanted to test if the selected Nbs were able to recognize the native ORF2 protein and ORF2 in the presence of 8 M urea. A 96-well microtiter plate was coated with 0.62 μg/ml of the ORF2 protein of the 1–4 HEV genotypes obtained under native conditions or in the presence of 8 M urea. As shown in Fig. 2D, Nb39 and Nb74 recognized only the soluble form of HEV-3 and HEV-4 ORF2 whereas HEV-1 and HEV-2 ORF2 were not recognized (Fig. 2D).

### Optimization of an immunoassay for the detection of antibodies against HEV-3 ORF2

**Figure 3:**
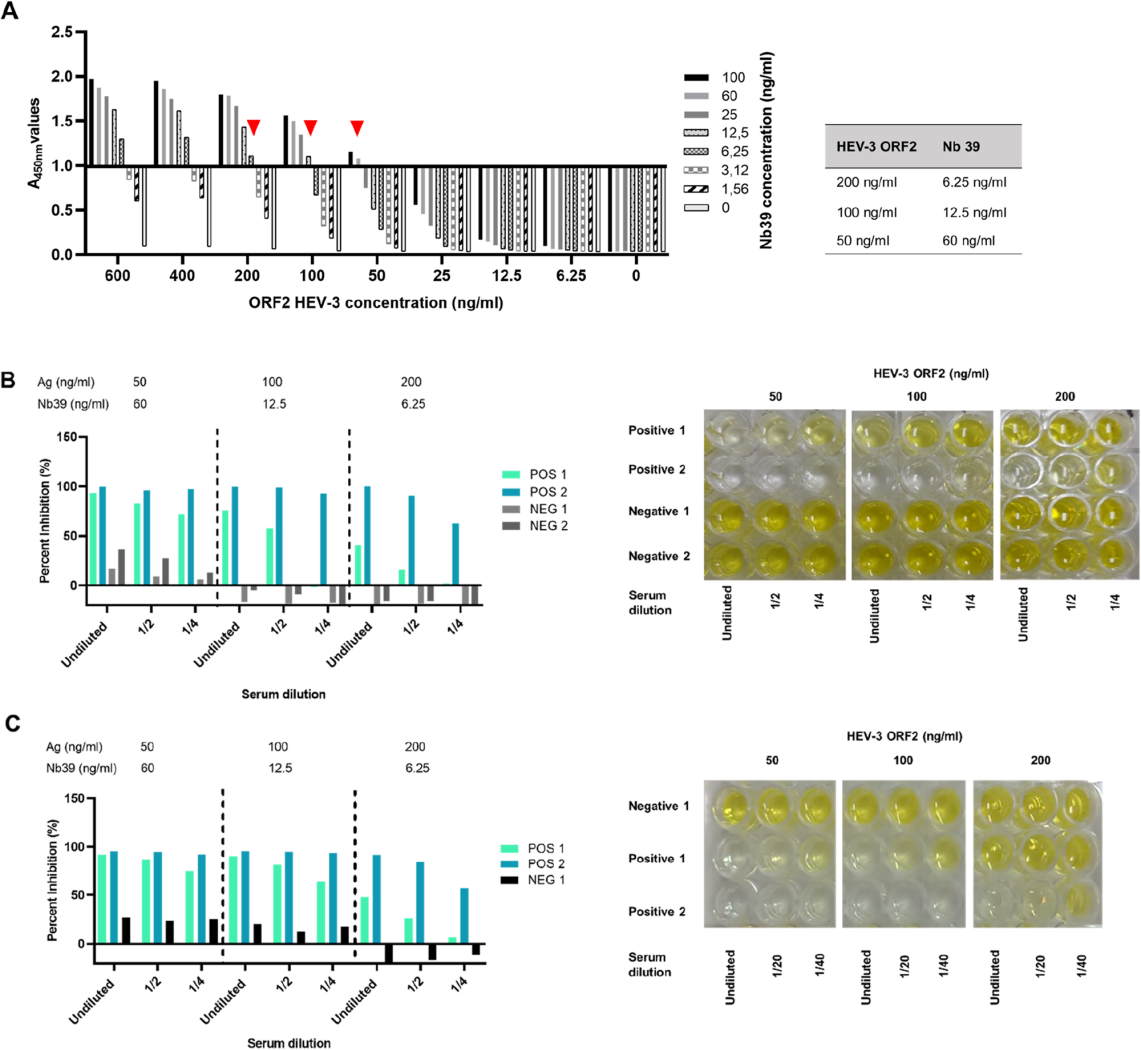
Parameter optimization of the cELISA. (**A**) iELISA. Selection of concentration of Ag and Nb. The figure shows the selected three points (red triangle) for Ag and Nb that can be observed on the right table. (**B**,**C**) cELISA. The optimal conditions of Ag, Nb, and serum dilution were selected. Human sera (**B**). Swine sera (**C**). The figure shows a lighter color in the positive serum using the following conditions: 50 ng/ml of Ag, undiluted serum, and 60 ng/ml of Nb39.

The optimal concentrations of antigen and the selected Nb39 were established by iELISA. 96-well microtiter ELISA plates were coated with different concentrations of HEV-3 ORF2 protein and after a blocking step, different concentrations of Nb39 were added. Figure 3A shows all the concentrations tested and the red arrows show the three paired HEV-3 ORF2-Nb39 values with an A_450nm_ = 1 that were selected for further analysis (Fig. 3A). Last, the optimal serum dilution was evaluated by cELISA using the three antigen-Nb39 concentration pairs selected before (200, 100, and 50 ng/ml of antigen and 6.25, 12.5, and 60 ng/ml of Nb39, respectively). As a result, the best conditions were: 50 ng/ml of HEV-3 ORF2 protein, undiluted serum, and 60 ng/ml of Nb39, considering the higher percent of inhibition (PI) of the positive serum with respect to the negative serum and the positive/negative (P/N) ratio as shown in Fig. 3B and Supplementary Table 1, respectively. Figure 3C and Supplementary Table 1 show that the same conditions used for humans are appropriate for evaluating domestic swine sera.

Summing up, the cELISA was established as follows: (a) 50 ng/ml HEV-3 ORF2 protein (100 μl/well) for 18 h at 4 °C; (b) blocking with 5% skimmed milk (200 μl/well) for 2 h at 37 °C; (c) undiluted serum (100 μl/well) for 1 h at 37 °C; (d) 5 washing steps (300 μl/well); (e) 60 ng/ml of Nb39 (100 μl/well); (f) washing five times (300 μl/well); (g) HRP anti-6xHis antibody (dilution 1:10,000) for 2 h at 37 °C, (h) 1 mg/ml of TMB (100 μl/well) for 15 min at RT; and (i) 1 M phosphoric acid (100 μl/well).

### Validation of the novel nanobody-based cELISA

**Figure 4:**
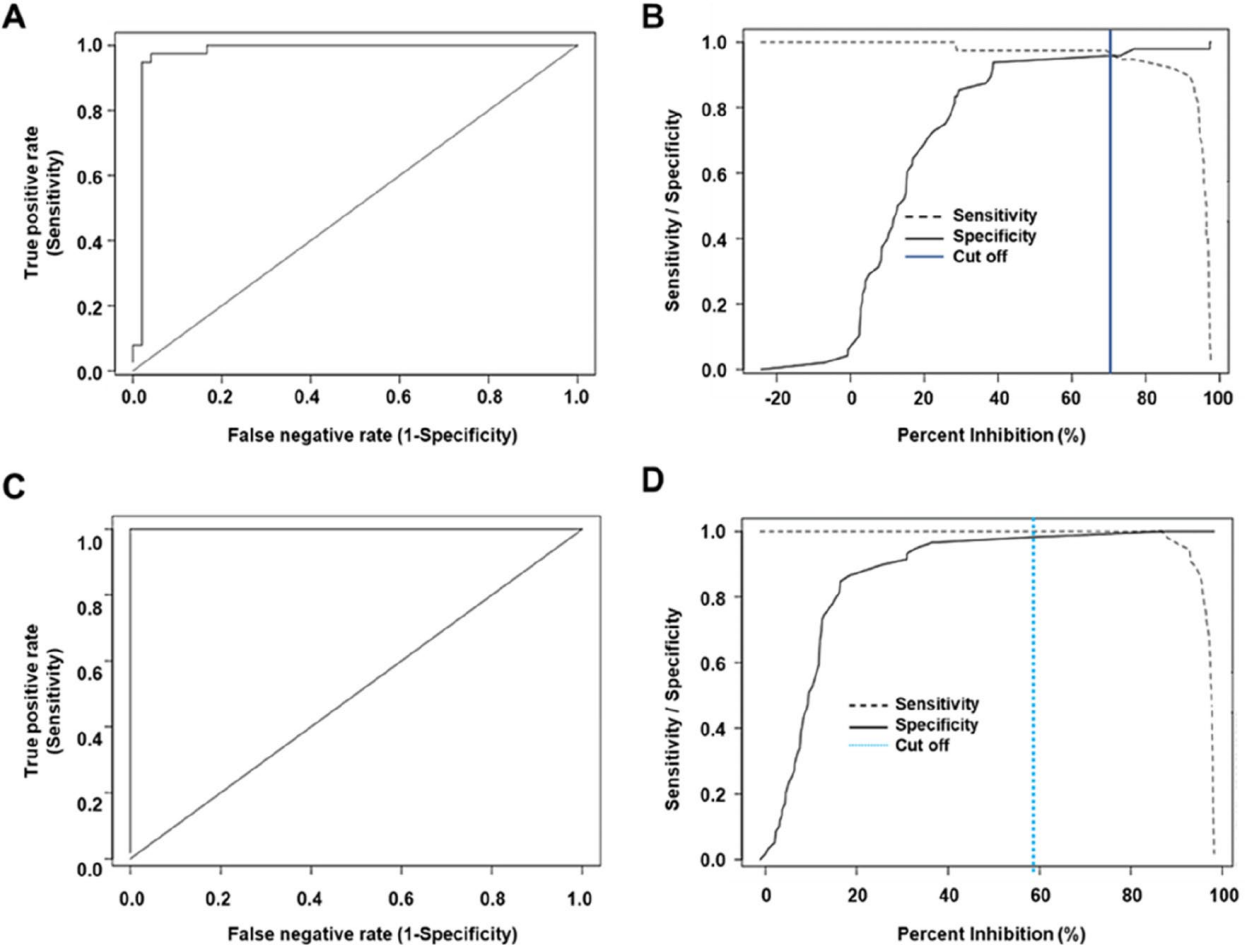
Determination of cutoff, sensitivity, and specificity for the cELISA. (**A**) Receiver operating characteristics (ROC) analysis shows sensitivity vs specificity for discrimination between positive and negative human serum samples that were screened for *in house* and commercial tests. AUC = 0.976. (**B**). The optimal values of the sensitivity and specificity curves were used to determine the cELISA cutoff = 69.16%, for human samples, full blue line. (**C**) ROC curve for swine serum samples. AUC = 1.000 (**D**) The cELISA cutoff for swine serum samples was 58.76%, dashed sky-blue line.

A total of 86 human and 116 domestic swine sera were tested with the cELISA. The individual PI values were calculated and then analyzed with the *Epitool* software. In the case of the human cELISA, the ROC curve is shown in Fig. 4A. The area under the curve (AUC) was 0.976 (95% CI 0.938–1). The ROC curve aided to set the cutoff value at 69.16% to determine the status of human serum samples (Fig. 4B). Therefore, a PI ≥ 69.16% is considered positive for human sera. The resulting data were compared with data obtained from a commercial ELISA (DIAPRO). The sensitivity and specificity were 97.4% (95% CI 86.5–99.5%) and 95,8% (95% CI 86.0–98.8%), respectively (Table 2).

**Table 2:** Validation of a novel *in house* cELISA.

Validation	Human	Swine
Cut off	69.16%	58.76%
Sensitivity	97.40% (95% CI 86.50–99.50%)	100% (95% CI 93.5–100%)
Specificity	95.80% (95% CI 86.00–98.80%)	98.30% (95% CI 91.00–99.70%)
Kappa index	0.93 (95% CI 0.85–1.00)	0.95 (95% CI 0.89–1.00)
AUC	0.98 (0.94–1.00)	1 (1–1)

In the case of the cELISA for swine sera, Fig. 4C shows the ROC curve with an AUC of 1 (95% CI 1–1). The cutoff value was 58.76%, indicating that a swine serum sample with a PI ≥ 58.76% is considered seropositive and below 58.76% is considered seronegative for HEV-3 (Fig. 4D). In this case, the sensitivity and specificity were 100% (95% CI 93.5–100%) and 98.3% (95% CI 91.0–99.7%), respectively (Table 2).

### Agreements of the developed nanobody-based cELISA with a commercial ELISA

For human serum samples (n = 86), the kappa index value of the cELISA with respect to the commercial immunoassay was 0.93 (95% CI 0.85–1.00). The 116 swine sera were tested with both the cELISA and the commercial kit, and the kappa index value was 0.95 (95% CI: 0.89–1.00), indicating in both cases an almost perfect agreement (Table 3).

**Table 3:** Kappa Index of novel cELISA for human and swine.

Human	Commercial ELISA			
cELISA		Positive	Negative	Total
	Positive	37	2	39
	Negative	1	46	47
	Total	38	48	86
k	0.93 (95% CI 0.85–1.00)			

Swine	Commercial ELISA			
cELISA		Positive	Negative	Total
	Positive	55	2	57
	Negative	1	58	59
	Total	56	60	116
k	0.95 (95% CI 0.89–1.00)			

### Reproducibility and detection limit

To analyze the reproducibility of the assay, 9 positive and negative human serum samples were tested by the cELISA to determine the intra-assay variability. Further, 2 positive and negative human sera were screened in the 6 different plates to evaluate the inter-assay variability. The intra-assay and inter-assay CV were < 10% and < 15%, respectively (Supplementary Table 2). These data indicated that the developed cELISA exhibited good reproducibility. The detection limit for the novel cELISA was ≥ 0.5 UI/ml (Supplementary Fig. S4).

### The novel cELISA is useful for detecting specific anti-HEV-3-ORF2 in several animal species

**Figure 5:**
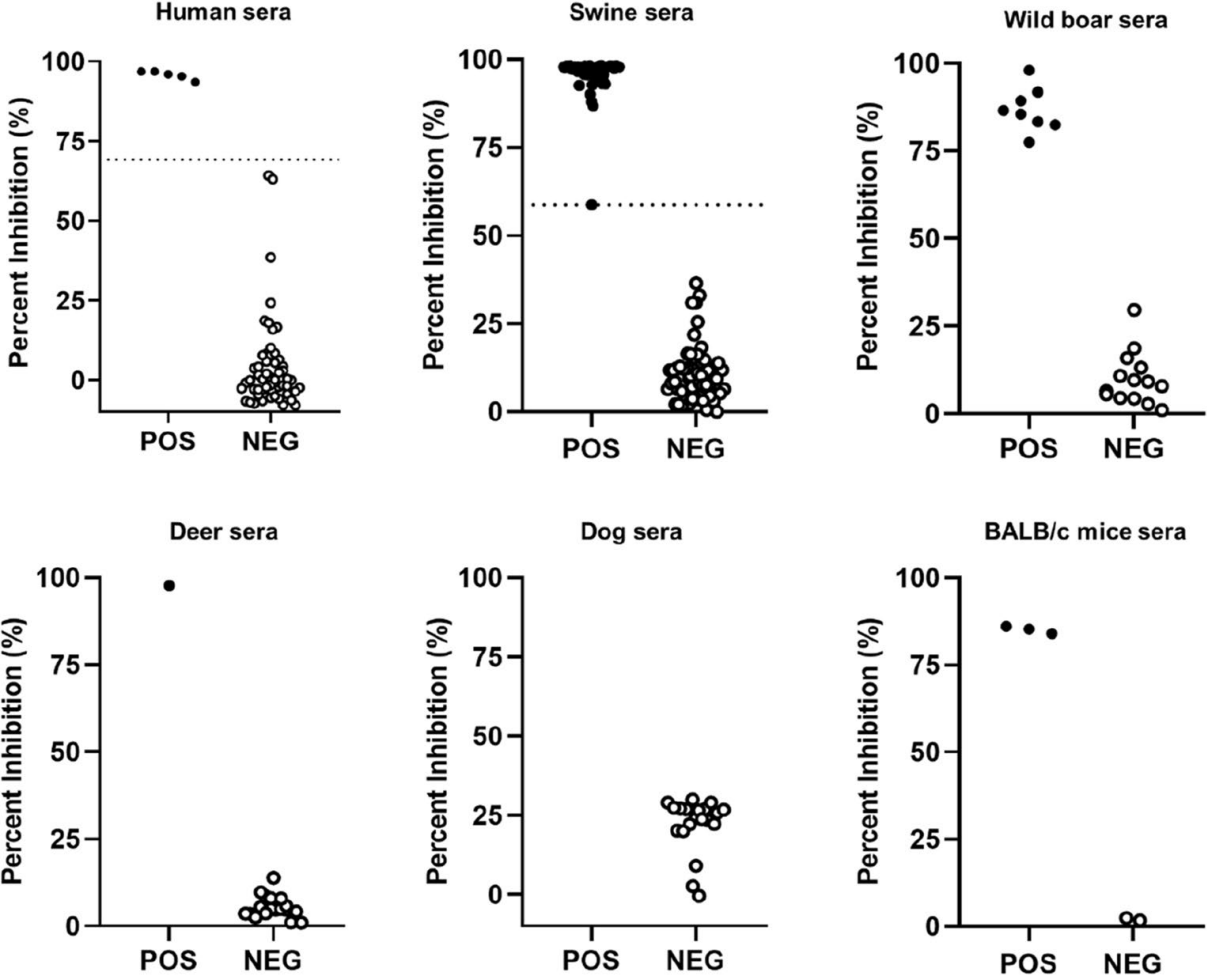
Detection of specific total anti-HEV antibodies with the novel cELISA in serum samples of different species (human, swine, wild boar, deer, dog, and mice).

To confirm whether the cELISA can be used to detect anti-HEV antibodies in serum samples from different animal species, we tested a total of 247 sera of different sample collections from swine, wild boar, deer, dogs, and mice, previously evaluated with commercial tests for HEV antibodies. The results are depicted in Fig. 5 and the characteristics of the samples are shown in the Supplementary Table 3. The results highly correlated with the commercial ELISA for all human and animal species tested except for dogs, one positive sample was not detected by the ELISA.

## Discussion

HEV is an emergent virus causing viral hepatitis worldwide^2^. There is an increasing concern as this virus has been isolated from different hosts with an ever-expanding host range, being the domestic pig and wild boar the most important reservoirs for genotypes 3 and 4^41^. As previously mentioned, in humans, HEV infection is generally self-limited and asymptomatic, but it can be fulminant in about 4% of the cases with an increased risk of 25% in pregnant women and the possibility to develop chronicity in immunocompromised patients^2,4,42^. HEV genotypes 3, 4, and 7 can be transmitted from animals to humans; thus, it is important to continue developing and improving diagnostic tests for this zoonotic disease^30^.

The HEV genome can be found in samples (blood or stool) for short periods of time during the early stages of infection. After that, humans and animals develop antibodies against the virus, which are detectable for a longer period of time^2^. Reverse transcription polymerase chain reaction and serological diagnostic methods are considered the main techniques to determine HEV infection^2^. The RT-PCR technique is expensive, complex, and it can be done only in laboratories where molecular biology know-how and specific equipment are available. In contrast, immunoassays such as ELISA, western blot, and immunofluorescence, can be used to detect viral particles and antibodies, their set-up is simpler, and the cost is lower^43–46^. Some of the serological diagnostic tests for humans have been adapted to detect antibodies in animals^33,34^. A disadvantage of these methods is that they require conventional antibodies (polyclonal or monoclonal antibodies) as their main reagents and their production is complex and expensive. The selection of hybridomas is a time-consuming and laborious process, it requires specialized laboratory techniques, and the cell lines must be stable through multiple cell generations, with high risk of losing a producing clone^47^. VHH, the variable domains of heavy chain-only antibodies of camelids and sharks, are small and very stable proteins that can be produced in bacterial and yeast expression systems with good yields. The microbial expression systems used for nanobody production are simpler and more cost-effective compared to hybridoma cell line maintenance. Nanobodies are highly versatile tools that have been used to detect different targets such as viral particles, toxins, hormones, antigens, and antibodies^43,48–50^. A great advantage of these antibody fragments is that they can be easily modified to generate capture and detection molecules stable at room temperature^51^. This is important considering the need of transporting the kits to areas where the maintenance of cold chain can be difficult.

ORF2, the capsid protein of HEV, is the main antigen towards which the immune response is directed^21^. Therefore, it is commonly used as antigen in several formats of immunoassays for detection of anti-HEV antibodies^21,52,53^. The glycosylated ORF2 (gORF2) is not part of the capsid and is released into plasma during viral replication^54^. The gORF2 can be present in sera for a longer time than the HEV genome and is a biomarker for active viral replication.

In this work, we selected two sets of Nbs with a variable germline origin. Two strategies of elution (trypsin and cellular) to obtain specific Nbs were applied, and we were able to obtain similar Nbs. For example, Nb14 and Nb57 sharing the same CDR3, were selected with cellular and trypsin elution, respectively (Table 1). The Nbs recognize the glycosylated form of the protein (data not shown) and are currently under study for their application in antigen detection assays. Six Nbs, selected considering their affinities and CDR3 sequences, were expressed and purified with good purity and yield from the periplasm of *E. coli* WK6, with a simple standard low-cost protocol^35,40^. The Nb39 and Nb74 were further selected for their higher affinities towards the HEV-3 ORF2 protein. Even though a neutralizing Nb against the HEV-4 ORF2 protein has been already described, the authors did not evaluate if it was able to recognize ORF2 from other genotypes^38^. In this study, we showed that both Nb39 and Nb74 were able to recognize ORF2 from 2 different genotypes: HEV-3 and HEV-4. Furthermore, these Nbs recognize a structural epitope, as they bound to ORF2 obtained only under native conditions in *E. coli* but not in the presence of 8 M urea (linear epitope) (Fig. 2D).

Hu et al. developed a double-antigen sandwich ELISA for detection of HEV-specific antibodies in human or swine sera^55^. The assay is based on the ORF2 protein as both capture and detection antigen, the latter conjugated with HRP. This process involves a periodate oxidation method, several steps of dialysis and a final step of stabilization. Other commercial tests, such as the one used in this study as reference, are also double-sandwich ELISA that use synthetic peptides for coating and HRP-labeled peptides for detection. Herein, we developed a cELISA which uses the recombinant HEV-3 ORF2 protein as capture antigen, the Nb39 to compete with the anti-HEV antibodies present in serum, and an anti-His antibody that recognizes bound Nbs as a detection reagent. Our novel cELISA detects specific antibodies in human, swine, wild boar, deer, and mouse sera. The use of undiluted serum samples results in a shorter procedure time and spares the use of a dilution reagent.

The novel cELISA showed high sensitivity, specificity, and almost perfect agreement with the commercial ELISA (DIAPRO) with kappa values of 0.93 (95% CI 0.85–1.00) for human and 0.95 (95% CI 0.89–1.00) for swine. Due to the number of available samples in each panel, the sensitivity and specificity were only calculated for human and swine sera obtaining excellent values of sensitivity [97.4% (95% CI 86.5–99.5%) for human and 100% (95% CI 93.5–100%) for swine] and specificity [95.8% (95% CI 86.0–98.8%) for human and 98.3% (95% CI 91.0–99.7% for swine. For the other species tested, the agreement with the commercial test was 100% except for dog samples. In this case there was only one positive sample available which could not be assessed as positive by our immunoassay. Further studies are needed to evaluate if this assay is suitable for dog samples, too.

In Argentina, the HEV is an emergent virus, which has not yet been included in the routine diagnosis of patients with liver pathology. In the present study, we screened 64 sera of patients with liver disease and found a seroprevalence of 8%. This result points out the need of including HEV in the routine diagnostic algorithm of viral hepatitis.

The assay for the detection of total immunoglobulins anti-hepatitis E virus has a significant practical utility in both clinic and community settings. On the one hand, this assay helps identifying individuals who have been exposed to hepatitis E virus and have developed an immune response through the production of immunoglobulins^2^. By detecting total immunoglobulins, the assay enables healthcare professionals to diagnose and monitor hepatitis E infections in patients. On the other hand, this assay provides valuable information for healthcare providers to assess the prevalence and incidence of hepatitis E virus infections among their patient population. The assay's sensitivity and specificity will contribute to its reliability in clinical settings, aiding in the implementation of appropriate patient management strategies, even in locations where diagnostics by molecular biology is unfeasible. In community settings, the assay's practical utility becomes even more apparent. It will allow for large-scale screening and surveillance programs to identify individuals who may have been exposed to hepatitis E virus within a community or population, such as blood donors^21^, professional occupations with animal reservoirs such as veterinarians, slaughterhouse and forestry workers, and hunters^56^. This information is crucial for public health officials to assess the spread of the virus, understand its epidemiology, and implement appropriate control measures. By detecting total immunoglobulins, the assay will help in identifying individuals and animal reservoirs with past or ongoing infections, even if they are asymptomatic or have mild symptoms.

In summary, we developed and validated a novel, multispecies, and specific cELISA to detect antibodies against HEV. The developed ELISA exhibited good sensitivity, specificity, and high consistency with the commercial kit. The cELISA can be used to test human clinical and veterinary serum samples. We have estimated that the local production cost of the biological components of the cELISA is at least 20 times lower than the cost of the imported kits.

To the best of our knowledge, this is the first report of production of Nbs against HEV-3 ORF2 for diagnostic purposes. These Nbs are useful tools that should be further investigated for their possible use in research and passive therapy, as it has been suggested for other virus infections^57,58^.

## Methods

### HEV-3 ORF2 recombinant protein production

The recombinant HEV-3 ORF2 protein (aa112–608) was used both as immunogen and as coating antigen, for the construction of a phage displayed Nb-library and development of the immunoassay^21^. The pETG-A-His-N-ORF2 plasmid was transformed into *E. coli* Rosetta by heat shock. Then, the selected clone was induced with 1 mM isopropyl β-d-1-thiogalactopyranoside (IPTG) at 37 °C for 4 h. After centrifugation, the bacteria were collected and resuspended in lysis buffer (10% glycerol, 20 mM Tris–HCl, 0.5 M NaCl, 5 mM imidazole, pH 7.9, supplemented with 0.02 mg/ml DNAse, 0.1% Triton, 0.2 mM PMSF, 1 mM DTT, and 1 mg/ml lysozyme). Finally, the N-terminal His-tagged protein was purified from the supernatant using a Ni–NTA resin (Thermo Fisher Scientific), the expression and purification of the HEV-3 ORF2 protein was checked by SDS-PAGE. The ORF2 protein of HEV-1, HEV-2, HEV-4 were expressed and purified in the same conditions to determine the binding capacity of selected Nbs.

### Llama immunization and library construction

Llama inoculation and sample collection were conducted by trained staff; this study was approved by the Animal Care and Use Committee of INTA (CICUAE) under the protocol N° 15/2020. A llama was intramuscularly injected with recombinant HEV-3 ORF2 protein (150 μg per dose) on days 0, 14, 28, and 50. Complete Freund’s adjuvant was used for the first dose and incomplete Freund’s adjuvant for the following boosts. The antibody responses were monitored by ELISA on serum samples taken after each immunization. For this, a 96-well microtiter plate was coated overnight with 100 ng of ORF2 protein per well in carbonate/bicarbonate buffer pH 9.6. Next day wells were blocked with 3% skimmed milk in PBST (PBS + 0.05% Tween20) for 1 h and llama serum, five-fold diluted in 1.5% skimmed milk, was added to the coated plates and incubated for 2 h at room temperature. After washing, 50 μl of HRP-linked anti-llama IgG (Bethyl Laboratories) diluted 1:15,000 was added. The reaction was developed with TMB and absorbance at 450 nm was measured (TECAN). The end point titer was estimated as the reciprocal of the highest dilution with an A_450_ that is at least double the A_450_ of preimmune serum sample.

Four days after the last boost, lymphocytes were isolated from 150 ml of anticoagulated blood using Ficoll Paque Plus (GE Life Sciences) in Leucosep tubes (GBO). Total RNA was extracted using a RNAeasy Midi kit (Qiagen) following the manufacturer's instructions. Complementary DNA was prepared with oligo(dT) primers using the First Strand cDNA Synthesis Kit (Roche). The VH and VHH genes were amplified using FastStart Taq DNA polymerase (Roche) using the following primers, CALL001: (5′-GTCCTGGCTGCTCTTCTACAAGG-3′) and CALL002: (5′-GGTACGTGCTGTTGAACTGTTCC-3′). A PCR amplicon of 0.7 kb was purified from the gel after electrophoresis using a Wizard^®^ SV Gel and PCR Clean-Up System (Promega). Purified PCR fragment was used as a template in a nested PCR to specifically amplify the VHH coding sequence using VHH-BACK-SAPI: (5′-CTTGGCTCTTCTGTGCAGCTGCAGGAGTCTGGRGGAGG-3′) and VHH-FORWARD-SAPI: (5′-TGATGCTCTTCCGCTGAGGAGACGGTGACCTGGGT-3′) primers. A Golden Gate assembly was done to clone the VHH sequences between two SapI sites of the pMECS-GG phagemid vector (containing coding sequences for HA and 6xHis tag at the C terminus), following a protocol previously described^59^. Electro-competent *E. coli* TG1 cells (Lucigen) were transformed with the purified ligation mixture and plated on selective agar medium. In order to determine the size of the library ten-fold serial dilutions of the recovered transformed cells were done in LB medium and later plated on selective agar medium. Individual colonies were picked to determine the number of clones containing VHH sequences. For this, a colony PCR was done using the following primers Fw-col: 5′-TTATGCTTCCGGCTCGTATG-3′; and Rv-col: 5′-CCACAGACAGCCCTCATAG-3′ as it has been described^40^.

### Biopanning of the nanobody-library against HEV-3 ORF2 protein

To select Nbs specific to HEV-3 ORF2 protein, 2 ml of bacteria from the stock library were inoculated into 2xTY medium until A_600nm_ = 0.6, afterwards the VCSM13 helper phage (Stratagene) was used to infect exponentially growing bacteria. The resulting Nb-library was panned three times on 96-well microtiter plates (Maxisorp Nunc) coated with the recombinant protein or the corresponding negative controls. The positive wells were coated with 0.2 μg of ORF2 in 100 μl PBS (phosphate-buffered saline) overnight at 4 °C, while the negative wells received only PBS. The next day, wells were washed twice with PBST and blocked with 2% skimmed milk in PBST. Approximately, 10^12^ phages were preincubated with 10 μl of blocking solution in 100 μl of PBS for 30 min at room temperature by head-over-head rotation, then added on positive and negative wells and incubated for 2 h on a vibrating platform (500 r.p.m.). During the first panning round wells were washed 10 times with PBST, while wells were washed 20 and 25 times for the second and third rounds respectively. Plates were incubated for 5 min on a vibrating platform (300 r.p.m.) every 5 washes. Specific phages were eluted with 100 μl of a 0.25 mg/ml trypsin solution (Sigma-Aldrich) for 30 min followed by neutralization with 5 μl of 4 mg/ml AEBSF solution (Carl Roth). A second elution step was done adding exponentially growing *E. coli* TG1 cells to the wells and incubating for 30 min at 37 °C. Trypsin eluted phages were amplified by infection of exponentially growing *E. coli* TG1 for 30 min. Infected TG1 cells were later superinfected with the VCSM13 helper phage. After superinfection, the culture was centrifuged at 2800*g* for 10 min at room temperature to remove traces of glucose and the pellet was resuspended in 2xTY medium supplemented with 100 μg/ml ampicillin and 25 μg/ml kanamycin. Next day, phages were purified using PEG 6000/NaCl precipitation and used for the next round of selection.

To obtain specific binders, individual TG1 colonies were screened by ELISA using periplasmic extract (PE-ELISA). For this, 95 colonies from the positive wells (from different panning rounds and elution strategies) and 1 colony from the negative well (colony 1) were inoculated in 1 ml of 2xTY medium containing 100 μg/ml ampicillin and 0.1% glucose in a 96-well deep well plates. Nbs expression was induced after incubation for 3 h at 37 °C and 200 r.p.m. with 1 mM IPTG (isopropyl β-d-1-thiogalactopyranoside). After induction for 4 h, cells were centrifuged, the pellet was frozen and thawed twice to disrupt cells, and resuspended in 120 μl of PBS. After centrifugation, the periplasmic extract was taken to determine specific binding of Nbs to the HEV-3 ORF2 protein.

### Screening of HEV-3 ORF2 protein-specific nanobodies

Specific binding of Nbs to the HEV-3 ORF2 protein was determined by ELISA. For this, 96-well microtiter plates (Maxisorp, Nunc) were coated overnight at 4 °C with 200 ng/well of recombinant protein or PBS as negative control. After 3 washes with PBST, the wells were blocked with 3% skimmed milk in PBST and 50 μl of the periplasmic extract was added to each well and incubated for 2 h at room temperature. After washing with PBST to remove excess of Nbs, specific binding was detected with an HRP-linked anti-HA antibody (Abcam) diluted 1:1500. After washing, 50 μl of TMB (3,3′,5,5′ tetramethylbenzidine, BD) was added, and the A450 nm was measured using a 96-well plate reader (TECAN). Plasmids were extracted from TG1 cells and transformed in *E. coli* DH5α in order to obtain high-quality DNA suitable for sequencing, plasmid DNA mini preparations were done using a commercial kit (Inbio Highway). Plasmids were sent for sequencing (Macrogen) to analyze their variability.

### Phylogenetic tree and germline analysis

Genetic similarity among sequenced Nbs was analyzed by a Neighbor-joining tree with 1000 bootstrap replicates using MEGA version 11. The IMGT/V-QUEST program (http://www.imgt.org/IMGT_vquest/vquest) was used to analyze the germline origin and to determine CDR3s of the selected Nbs. Nanobody sequences were also analyzed by IMGT/DomainGapAlign (https://www.imgt.org/3Dstructure-DB/cgi/DomainGapAlign.cgi). Sequence logo was plotted using WebLogo3^60^.

### Expression and purification of nanobodies in *E. coli* WK6

Plasmids encoding the selected Nbs were transformed into *E. coli* WK6 by heat shock and 2 colonies from each construct were inoculated in 1 ml of LB medium supplemented with 100 μg/ml ampicillin and 2% glucose for 18 h at 37 °C and 200 r.p.m. Then, 30 μl of the culture was added to 3 ml of TB medium supplemented with 100 μg/ml ampicillin, 0.1% glucose, and 1 mM MgCl_2_ and incubated for 2 h 30 min at 37 °C and 200 r.p.m. Protein expression was induced with 1 mM IPTG for 4 h at 37 °C. Then, the cell pellet was mixed with 45 μl of ice-cold TES buffer (0.2 M Tris–HCl, 0.5 mM EDTA, 0.5 M sucrose), after incubating for 1 h on ice, 130 μl of H_2_O_dd_ were added and further incubated for 2 h. Finally, the periplasmic extract was obtained by centrifugation at 4 °C and analyzed by Western Blot^61^. For scaled-up expression, 2 ml of the overnight culture were added to 200 ml of culture medium (TB with ampicillin, glucose and MgCl_2_) at 37 °C and 200 r.p.m. When the culture reached an A_600nm_ = 0.6, protein expression was induced with 1 mM IPTG at 28 °C overnight. After centrifugation, the periplasmic proteins were extracted by osmotic shock following a protocol described elsewhere^61^. C-terminal 6xHis tagged Nbs were purified by affinity chromatography (Ni–NTA resin) and identified by SDS-PAGE. The concentration of Nbs was determined by the Bradford method.

### Optimization and development of cELISA using anti-HEV-3 ORF2 protein-specific nanobodies

After selecting the Nbs, we proceeded with the development of a cELISA as follows. A 96-well microtiter plate (JetBiofil^®^) was coated with 100 μl of HEV-3 ORF2 protein per well (0.62 and 1.25 μg/ml) and incubated for 18 h at 4 °C. Wells were blocked with 200 μl of 5% skimmed milk during 2 h at 37 °C. Subsequently, different concentrations of Nbs (1; 0.5; 0.25; 0.12; 0.062 and 0.031 μg/ml) were added and incubated for 1 h at 37 °C. After washing 3 times with PBS, 100 μl HRP-linked anti-6xHis antibody (Abcam) that recognizes only the C-terminal 6xHis tag, diluted 1:10,000, was added for 1 h at 37 °C. Finally, 100 μl of 0.1 mg/ml TMB (Sigma) was added for 15 min at room temperature, the color reaction was stopped with 1 M phosphoric acid, and the A_450nm_ was measured using an ELISA plate reader (Allshen, China). The Nbs with the best affinity were re-tested at lower concentrations (0.06; 0.03; 0.015; 0.008; 0.004, 0.002; 0.001; and 0 μg/ml) by indirect ELISA (iELISA).

Different parameters were optimized including antigen concentration, Nb concentration, and serum dilution. To optimize the concentrations of the antigen and Nb, an iELISA was employed. In this experiment, 100 μl of HEV-3 ORF2 protein was coated onto a 96-well microtiter plate at various concentrations (600, 400, 200, 100, 50, 25, 12.5, 6.25, and 0 ng/ml), and the plate was left to incubate at 4 °C for 18 h. Following blocking with 5% skimmed milk, 100 μl of Nbs at different concentrations (100, 60, 25, 12.5, 6.25, 3.12, and 1.56 ng/ml) were added and incubated for 1 h at 37 °C. The optimal conditions for HEV-3 ORF2 protein and Nbs were determined based on an A_450nm_ value close to 1.0.

The dilution of positive and negative sera from both human and pig samples was optimized for the development of the cELISA. For this, the HEV-3 ORF2 protein was coated overnight at 4 °C. Then, 100 μl of the respective serum dilution (undiluted, 1/2, and 1/4) were added and incubated for 1 h at 37 °C. After washing, 100 μl of Nbs were added and incubated for 1 h at 37 °C. Then, 100 μl of HRP-linked anti-6xHis antibody was added for 1 h at 37 °C. After each incubation, wells were washed 5 times with PBST. Finally, the TMB substrate was added and incubated for 15 min at room temperature. One molar phosphoric acid was used to stop the reaction. The optimal serum dilution was determined considering the percent of inhibition (PI) and the minimum ratio between the A_450nm_ value of a positive and a negative serum (P/N). Lastly, the cELISA was established as follow: a 96-well microtiter plate was coated with the optimal concentration of the HEV-3 ORF2 protein (100 μl/well) and incubated overnight at 4 °C. After washing with PBS, the plate was blocked with 5% skimmed milk (200 μl/well) for 2 h at 37 °C. After washing, the serum dilution (100 μl/well) was added and incubated for 1 h at 37 °C. The optimal dilutions of Nbs (100 μl/well) were added and incubated during 1 h at 37 °C. After washing, a HRP-linked anti-6xHis antibody (100 μl/well) was added for 1 h at 37 °C and after washing 0.1 mg/ml TMB (100 μl/well) was added and incubated at 37 °C in the dark for 15 min. The reaction was stopped with 1 M phosphoric acid and the A_450 nm_ values were read and converted to percent of inhibition (PI) using the formula: PI = 100 − (A_450nm_ tested sample/A_450nm_ negative control) × 100%^62^. As a negative control for the cELISA, buffer was added instead of serum. There was no cross reactivity of the HRP-linked anti-6xHis antibody with the N-terminal-His-tagged ORF2 (results not shown).

### Validation of cELISA and comparison with a commercial ELISA

Eighty-six human and 116 swine sera were confirmed as positive for HEV by an iELISA (Diapro, Italy) or by an antigen double sandwich ELISA (Diapro, Italy), respectively. Then, both sera panels were tested with the cELISA. All data were analyzed with *Epitool* software to determine the cut off values with the receiver operating characteristic (ROC) curve, the area under the curve (AUC), sensitivity, and specificity. The k index value was also calculated to determine the agreement between the commercial and the novel cELISA.

To evaluate the reproducibility of the developed cELISA, 9 positive and 9 negative human sera were tested, each serum was analyzed in 9 replicates on the same 96-well microtiter plate. Then, two positive and 2 negative human sera were tested on 6 plates at different times to calculate the coefficient of variation (CV), and to evaluate the inter-assay (between plates) and intra-assay variabilities (within a plate).

The limit of detection was determined with a standard serum for HEV antibodies (1 UI/ml) (Diapro, Italy). The serum was diluted (1, 0.5, 0.25, 0.125, and 0.062 UI/ml) and then tested with the cELISA.

### Screening of total antibodies anti-HEV-3 ORF2 protein in different species

The cELISA was used to test several samples of human and animal sera to assess the possibility that our assay could be used to detect anti-HEV antibodies in different species. Human samples comprised a panel of 64 sera of patients with liver disease which were collected in 2019 (CEI 27-2019, ethic committee of the provincial system of health (SIPROSA), Tucumán, Argentina). Informed consent was obtained from all participants. Swine (n = 116), wild boar (n = 22), deer (n = 20), and dog (n = 20) samples are part of a serum collection obtained during 2017–2022. The samples from BALB/c mice (n = 5) were obtained after a subcutaneous immunization of mice every 3 weeks with the recombinant HEV-3 ORF2 protein (CICUAL-UNT N°025/2019). All sera were pre-screened using two different commercial kits: the antigen double sandwich ELISA (Diapro, Italy) for animal samples and the total Ig indirect ELISA (Diapro, Italy) for human samples. The serum samples were stored at − 20 °C.

### Statistical analysis

Data was analyzed with GraphPad Prism version 8.0 (GraphPad Software, San Diego, CA, USA). The validation and k index value were evaluated using Epitool software (https://epitools.ausvet.com.au/). The CV values less than 10% and 15% were considered an acceptable repeatability level for the intra-plate assay and inter-assay variabilities, respectively^21^.

### Ethical approval

All methods were carried out in accordance with relevant guidelines and regulations. Animal immunizations were performed in strict accordance with the ARRIVE guidelines and were approved by the Ethical Committee of the National University of Tucumán (CICUAL-UNT) Nr.043/2021.

### Supplementary Information

Supplementary Information.

## Data Availability

Data are available from the corresponding authors (Lorena Itatí Ibañez and María Guadalupe Vizoso Pinto) upon reasonable request.
