# Supplementary material for: A multispecies competitive nanobody-based ELISA for the detection of antibodies against hepatitis E virus
Source: Sci Rep. 2023 Sep 18;13:15448. doi: 10.1038/s41598-023-41955-z (PMC10507121; doi:10.1038/s41598-023-41955-z)
Supplement: Supplementary file 1 — Supplementary Information. [file 41598_2023_41955_MOESM1_ESM.pdf]

A MULTISPECIES COMPETITIVE NANOBODY-BASED ELISA FOR THE DETECTION OF ANTIBODIES AGAINST HEPATITIS E VIRUS

Lorena Paola Arce[1,2], María Florencia Pavan[2], Marina Bok[3], Silvina Elena Gutiérrez[4], Silvia Marcela Estein[4], Agostina Tammone Santos[4]; Walter Ezequiel Condorí[4]; Marcela María Uhart[5], Viviana Parreño[3], María Guadalupe Vizoso-Pinto[1,+,*] & Lorena Itatí Ibañez[2,+,*]

[1] Infection Biology Laboratory, Faculty of Medicine and Instituto Superior de Investigaciones Biológicas (INSIBIO), CONICET-UNT, Tucumán, T4000ILI, Argentina

[2] Laboratorio de Ingeniería de Anticuerpos. Instituto de Química, Física de los Materiales, Medio ambiente y Energía (INQUIMAE- CONICET), Ciudad Autónoma de Buenos Aires, C1428EGA, Argentina

[3] InculNTA, Instituto de Virología, Instituto Nacional de Tecnología Agropecuaria (INTA), Husrlingham, 1686, Argentina

[4] Universidad Nacional del Centro de la Provincia de Buenos Aires, Facultad de Ciencias Veterinarias, Núcleo SAMP. Centro de Investigación Veterinaria de Tandil (UNCPBA-CICPBA-CONICET), Tandil, Buenos Aires, B7000GHG, Argentina

[5] One Health Institute, School of Veterinary Medicine, University of California Davis, Davis 95616, California, USA

*Correspondence to: Dr. María Guadalupe Vizoso-Pinto Email: mgvizoso@fm.unt.edu.ar

*Correspondence to: Dr. Lorena Itatí Ibañez Email: loreitati@gmail.com

[+] These authors contributed equally to this work

Supplementary Information

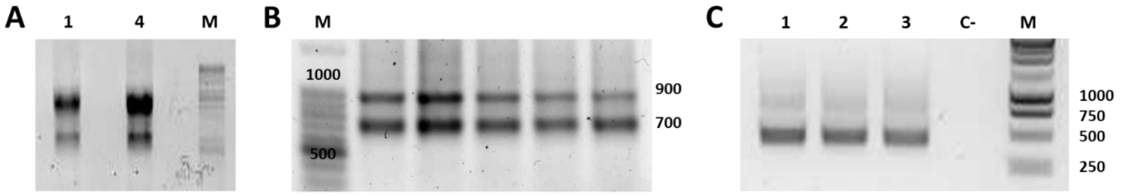

Supplementary Figure S1. Construction of an immune Nb library. (**A**) Total RNA extracted from llama lymphocytes (sample 1 and 4) was reversed transcribed into cDNA and used as templates for the nested PCR. (**B**) First PCR, a fragment of ~700 bp representing the heavy chain–only antibody repertoire and a fragment of 1,000 bp corresponding to the heavy chain of the conventional antibodies were obtained. (**C**) The band of 700 bp was isolated and used as template for the second PCR, a fragment of 400 bp, corresponding to the VHHs was observed.

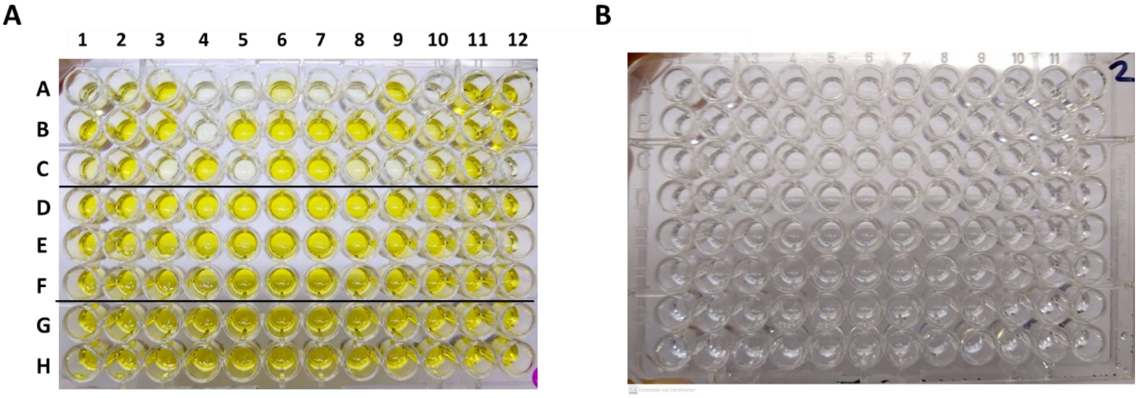

Supplementary Figure S2. Screening of specific Nbs against HEV-3 ORF2 protein extracted from periplasm of infected TG1 bacteria. **(A)** ELISA on a plate coated HEV-3 ORF2 protein. **(B)** ELISA on a plate coated with an irrelevant protein (negative control). Specific reaction was developed using horseradish peroxidase (HRP)-linked anti-HA antibody and the TMB substrate. Colonies were picked from agar plates seeded with infected TG1 after different round of panning and elution strategies as follows: A1-A12 and B1-B4 (first round of panning - cell elution); B5-B12 and C1-C12 (first round of panning - trypsin elution); D1-D12 and E1-E4 (second round of panning - cell elution); E5-E12 and F1-F12 (second round of panning - trypsin elution); G1-G12 and H1-H6 (third round of panning - trypsin elution) and H6-H12 (third round of panning – cell elution).

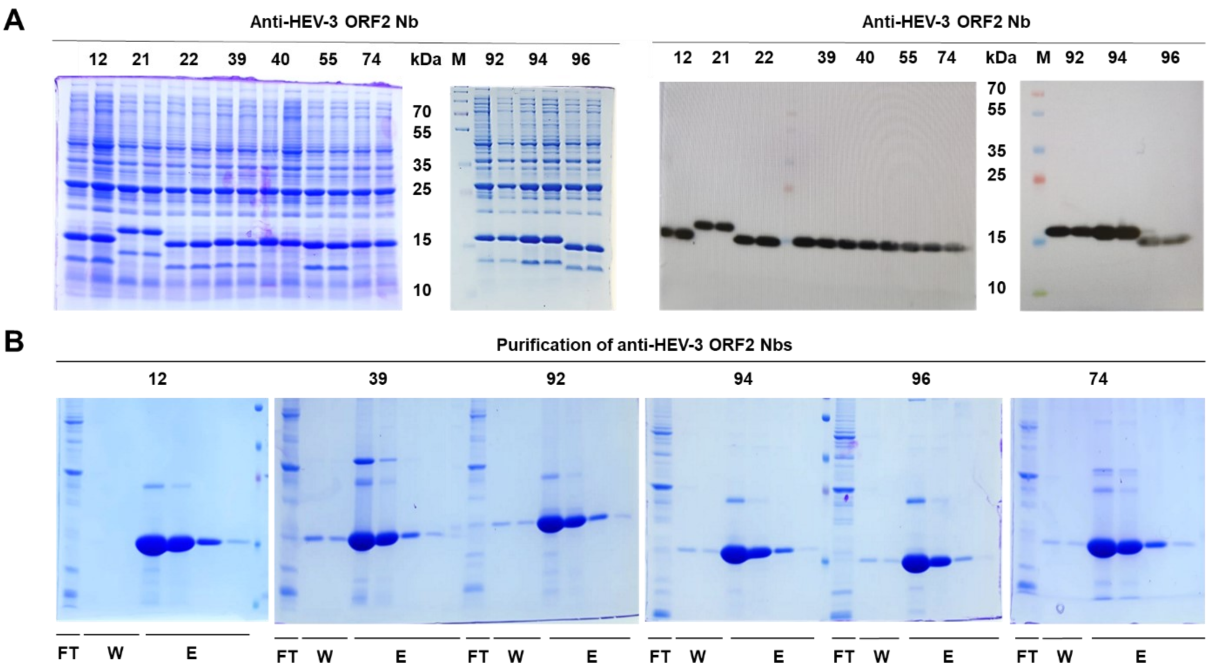

Supplementary Figure S3. Expression and purification of anti-HEV-3 ORF2 Nbs. **(A)** Ten Nbs (two different clones for each Nb) were analyzed by SDS-PAGE and Western blot, proteins with small differences in size were observed. **(B)** Purification of 6 Nbs by affinity chromatography (Ni-NTA resin), FT: flow through, W: wash, and E: elution.

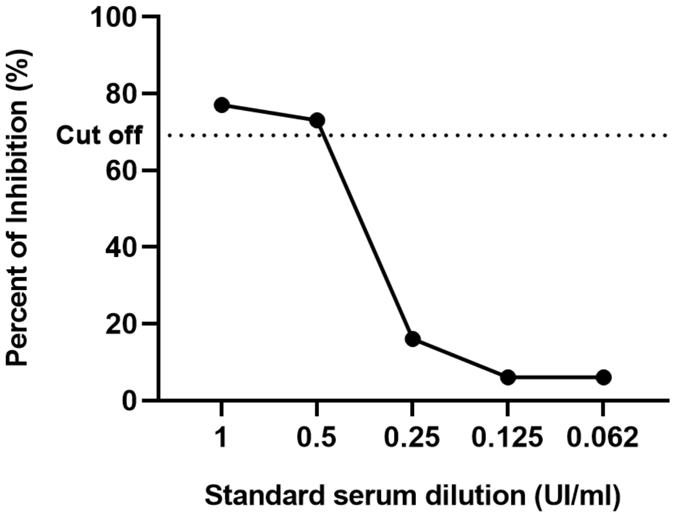

Supplementary Figure S4. Limit of detection of the cELISA. Standard serum for HEV antibodies was diluted and analyzed by developed cELISA.

Supplementary Table 1 Determination of the positive/negative ratio to select the optimal conditions of a novel cELISA for human and swine sera

Antigen HEV-3 ORF2	0.05 ug/ml			0.1 ug/ml			0.2 ug/ml		
Serum dilution	Undiluted	1/2	1/4	Undiluted	1/2	1/4	Undiluted	1/2	1/4
Human									
Positive 1	0.097	0.264	0.424	0.363	0.639	1.546	0.900	1.277	1.487
Negative 1	1.264	1.374	1.424	1.770	1.795	1.783	1.869	1.793	1.866
P/N	0.077	0.192	0.298	0.205	0.356	0.867	0.482	0.712	0.797
Positive 2	0.004	0.057	0.040	0.001	0.011	0.108	-0.001	0.143	0.567
Negative 2	0.966	1.103	1.316	1.592	1.649	1.799	1.755	1.756	1.858
P/N	0.005	0.052	0.031	0.001	0.007	0.06	-0.001	0.081	0.305
Swine									
Positive 1	0.128	0.201	0.384	0.151	0.288	0.548	0.794	1.122	1.417
Negative 1	1.111	1.162	1.136	1.214	1.326	1.254	1.813	1.774	1.686
P/N	0.115	0.173	0.338	0.124	0.217	0.437	0.438	0.632	0.840
Positive 2	0.069	0.081	0.123	0,068	0,074	0,098	0.132	0.249	0.654
Negative 1	1.111	1.162	1.136	1.214	1.326	1.254	1.813	1.774	1.686
P/N	0.062	0.070	0.108	0.056	0.056	0.078	0.073	0.140	0.387

Supplementary Table 2 Reproducibility of novel cELISA

Intra-assay coefficient of variance (CV)							
Serum	Abs 1	Abs 2	Abs 3	Abs 4	Mean	SD	CV%
Positive 1	0.472	0.473	0.464	0.476	0.471	0.010	1.090
Positive 2	0.136	0.137	0.130	0.141	0.136	0.000	3.340
Positive 3	0.051	0.054	0.054	0.054	0.053	0.000	2.820
Positive 4	0.039	0.039	0.042	0.039	0.039	0.000	3.770
Positive 5	0.045	0.045	0.044	0.044	0.044	0.000	1.300
Positive 6	0.048	0.044	0.046	0.044	0.045	0.000	4.210
Positive 7	0.201	0.198	0.204	0.198	0.200	0.000	1.430
Positive 8	0.051	0.048	0.052	0.048	0.049	0.000	4.140
Positive 9	0.041	0.045	0.046	0.042	0.043	0.000	5.470
Negative1	1.695	1.544	1.749	No data	1.662	0.110	6.390
Negative 2	1.759	1.686	1.812	No data	1.752	0.060	3.610
Negative 3	1.860	1.774	1.736	No data	1.790	0.060	3.550
Negative 4	1.643	1.581	1.581	1.513	1.579	0.050	3.360
Negative 5	1.533	1.718	1.601	1.666	1.629	0.080	4.920
Negative 6	1.612	1.632	1.688	No data	1.644	0.040	2.400
Negative 7	1.653	1.766	1.847	1.742	1.752	0.080	4.560
Negative 8	1.495	1.444	1.513	1.447	1.474	0.030	2.350
Negative 9	1.624	1.617	1.581	No data	1.607	0.020	1.440

Inter-assay coefficient of variance (CV)									
Serum	Abs 1	Abs 2	Abs 3	Abs 4	Abs 5	Abs 6	Mean	SD	CV%
Positive 1	0.145	0.160	0.137	0.124	0.127	0.146	0.139	0.010	9.570
Positive 2	0.052	0.062	0.064	0.055	0.067	0.068	0.061	0.010	10.600
Negative1	1.812	1.860	1.825	1.782	1.792	1.856	1.821	0.030	1.770
Negative 2	1.014	1.250	1.345	1.285	1.26	1.02	1.195	0.150	12.130

Supplementary Table 3 Agreements of the cELISA with a commercial ELISA with samples from different species

		Commercial ELISA		
		Positive	Negative	Total
**cELISA (human)**	Positive	5	0	5
	Negative	0	59	59
	Total	5	59	64
	Sensitivity 100 %			
	Specificity 100 %			
**cELISA (swine)**	Positive	55	2	57
	Negative	1	58	59
	Total	56	60	116
	Sensitivity 98.21 %			
	Specificity 96.67 %			
**cELISA (wild boar)**	Positive	8	0	8
	Negative	0	14	14
	Total	8	14	22
	Sensitivity 100 %			
	Specificity 100 %			
**cELISA (deer)**	Positive	1	0	1
	Negative	0	19	19
	Total	1	19	20
	Sensitivity 100 %			
	Specificity 100 %			
**cELISA (dog)**	Positive	0	0	0
	Negative	1	19	20
	Total	1	19	20
	Sensitivity 0 %			
	Specificity 100 %			
**cELISA (mice)**	Positive	3	0	3
	Negative	0	2	2
	Total	3	2	5
	Sensitivity 100 %			
	Specificity 100 %			

Unmodified gels

Supplementary Figure S1.

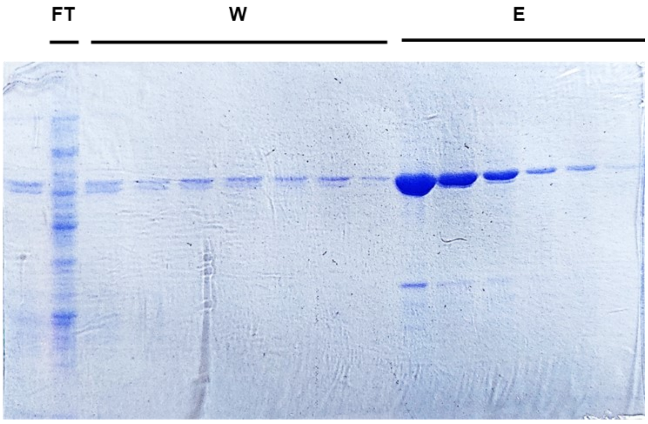

(**A**) SDS-PAGE and Coomassie blue staining showing the expression and purification of the recombinant HEV-3 ORF2 protein. FT: flow through, W: wash, and E: elution.

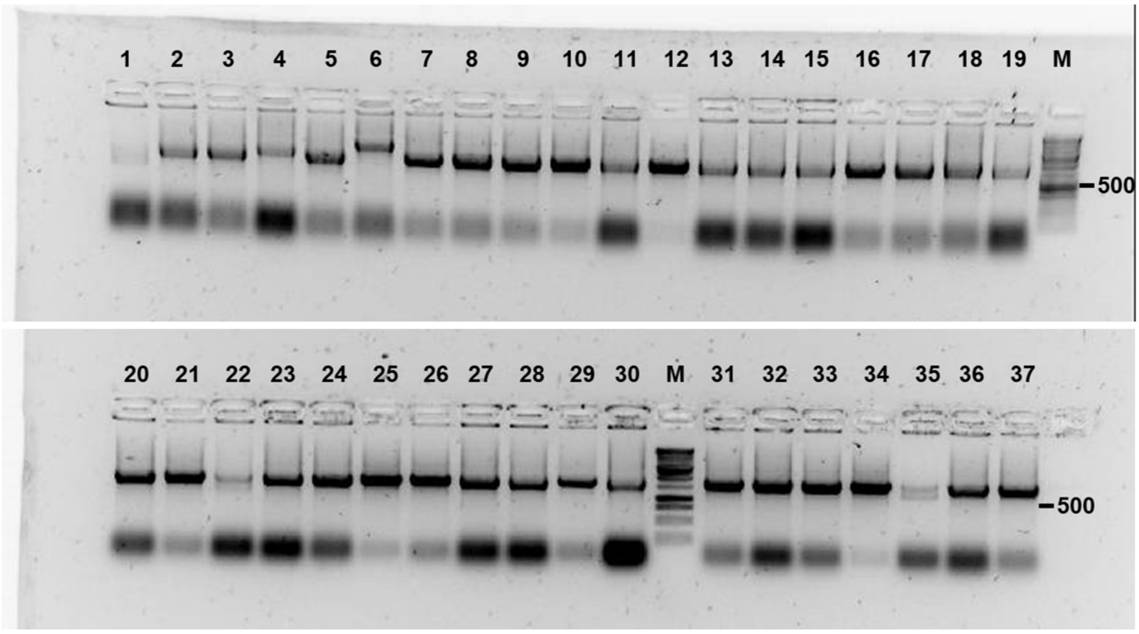

(**D**) Agarose gel showing the ~700 bp PCR fragments corresponding to VHHs of variable size amplified from randomly picked individual colonies.

Supplementary Figure S2.

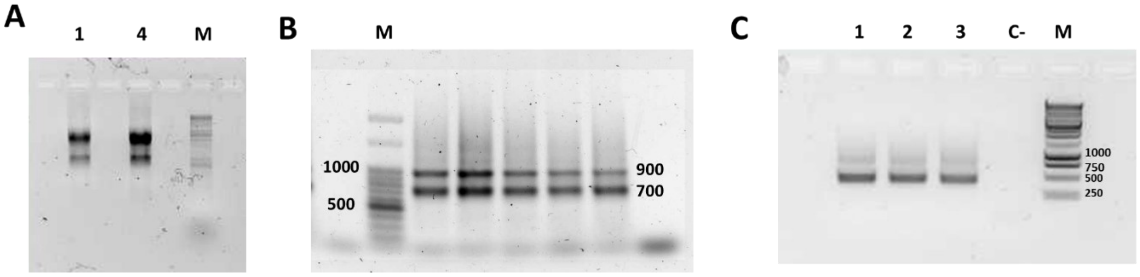

(**A**) Total RNA extracted from llama lymphocytes (sample 1 and 4) was reversed transcribed into cDNA and used as templates for the nested PCR.

(**B**) First PCR, a fragment of ~700 bp representing the heavy chain–only antibody repertoire and a fragment of 1,000 bp corresponding to the heavy chain of the conventional antibodies were obtained.

(**C**) The band of 700 bp was isolated and used as template for the second PCR, a fragment of 400 bp, corresponding to the VHHs was observed.

Supplementary Figure S4.

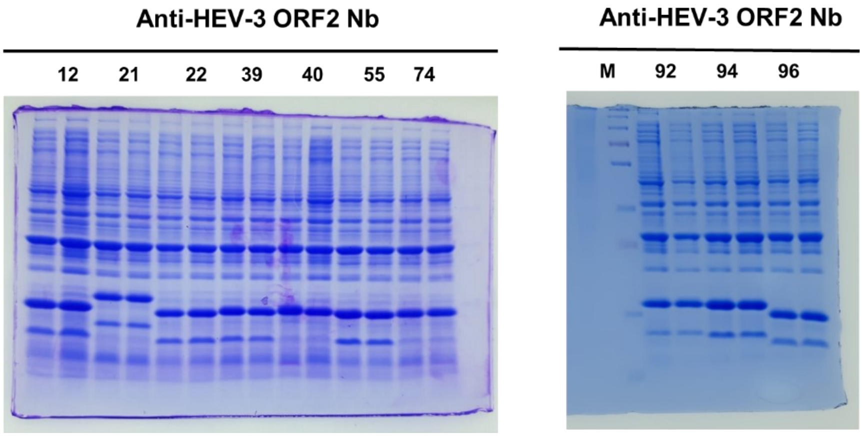

(**A**) Ten Nbs (two different clones for each Nb) were analyzed by SDS-PAGE and Western blot, proteins with small differences in size were observed.

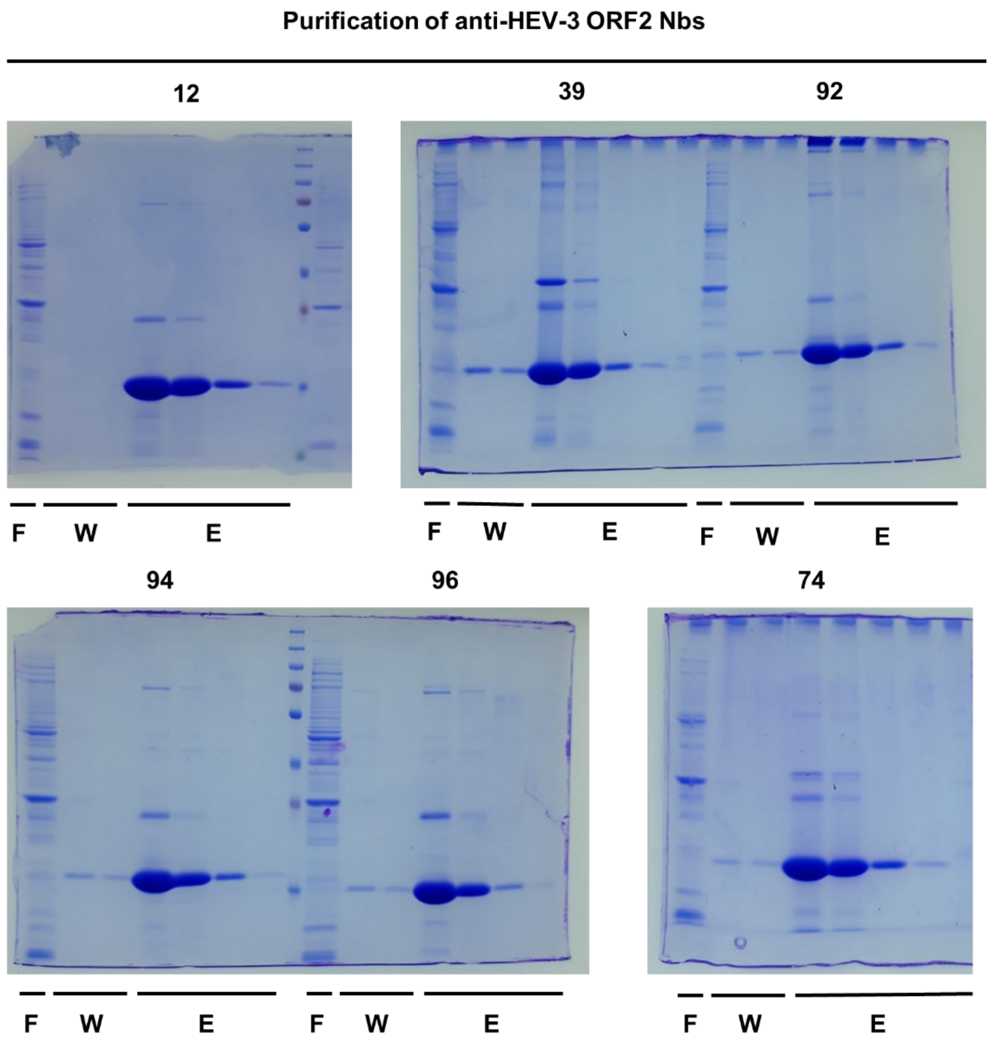

(B) Purification of 6 Nbs by affinity chromatography (Ni-NTA resin), FT: flow through, W: wash, and E: elution.
